# A blood-based four-gene diagnostic signature for Kashin–Beck disease revealed by multi-cohort transcriptomic analysis and machine learning

**DOI:** 10.3389/fimmu.2026.1789022

**Published:** 2026-05-13

**Authors:** Minghui Guo, Kunkun Yang, Shizhang Liu, Zhengming Sun, Xueyuan Wu, Xinpei Li, Yuchao Wang, Xinke Zhu, Ming Ling

**Affiliations:** 1 Department of Orthopaedics, Shaanxi Provincial People’s Hospital, Xi’an, China; 2 Key Laboratory of Basic and Clinical Translational of Bone and Joint Diseases in Shaanxi Provincial, Xi’an, China

**Keywords:** blood transcriptomics, gene signature, immune cell composition, Kashin–Beck disease, machine learning

## Abstract

**Background:**

Kashin–Beck disease (KBD) is an endemic osteoarthropathy characterized by growth retardation and progressive joint degeneration. However, its systemic molecular features in peripheral blood remain incompletely understood.

**Methods:**

Peripheral blood transcriptomic data from four independent cohorts were analyzed using differential expression analysis and weighted gene co-expression network analysis to identify KBD-associated gene sets. Multiple feature selection strategies and machine learning models were applied to construct and validate a blood-based diagnostic signature across cohorts. Immune cell composition was inferred by computational deconvolution, and transcription factor regulation, pathway enrichment, and genetic association data were integrated for biological interpretation.

**Results:**

A four-gene blood signature (*C4B*, *AQP1*, *HBA2*, and *ACSL6*) was identified, showing stable diagnostic performance across independent blood cohorts and preserved discriminatory capacity in cartilage tissue. Downstream analyses revealed that the diagnostic genes were associated with altered immune cell composition and immune- and metabolism-related pathways in peripheral blood.

**Conclusions:**

This study defines a compact and interpretable blood-based transcriptomic signature for KBD and provides insight into its systemic immune-related molecular context, supporting its potential utility for disease identification and mechanistic investigation.

## 1 Introduction

Kashin–Beck disease (KBD) is an endemic and chronic osteochondral disorder. It is characterized by growth plate cartilage necrosis, epiphyseal deformity, and progressive joint degeneration (1, 2). Patients typically present with finger enlargement, brachydactyly, and joint deformities. Severe cases may progress to dwarfism and lifelong disability, leading to marked impairment of quality of life (3). Although the incidence of new pediatric cases has declined after nutritional and environmental interventions, many adults continue to suffer from residual skeletal deformities and degenerative joint disease (4). Current diagnosis and disease grading mainly rely on clinical manifestations and radiographic findings of affected joints. These structural changes usually appear after irreversible cartilage and subchondral bone damage, limiting early disease identification.

High-throughput sequencing technologies have improved the understanding of KBD at the tissue level (5–7). Transcriptomic analyses of articular cartilage have identified genes and pathways that distinguish KBD from primary osteoarthritis. These studies highlighted abnormalities in extracellular matrix remodeling, chondrocyte apoptosis, and growth plate development (8). Multi-omics studies further implicated selenoprotein networks, epigenetic regulation, and RNA methylation in KBD pathogenesis (9–12). Several candidate genes, including ADAMTS14, SLC13A5, CEACAM1, and RUNX2-related pathways, have been proposed as potential biomarkers (6). However, cartilage and subchondral bone samples are difficult to obtain from living individuals, which limits their use in large-scale screening and longitudinal studies.

Peripheral blood provides an accessible alternative for molecular investigation. Early blood transcriptome studies reported widespread gene-expression changes in KBD, including alterations in immune-related pathways (5, 13). Comparative analyses across disease stages and between KBD and osteoarthritis identified differentially expressed genes and proposed preliminary blood-based diagnostic panels (14). Subsequent studies combining blood gene expression with clinical data suggested associations between specific genes and disease severity (15). Serum metabolomic studies also revealed altered amino acid and lipid profiles in patients with KBD (16, 17). These findings indicate that blood-based molecular signatures may reflect systemic features of the disease. However, most previous studies relied on single cohorts, limited feature-selection strategies, and single modeling approaches. Cross-cohort validation and model interpretability were often lacking.

The present study aimed to identify a stable and interpretable blood-based molecular signature for KBD using a multi-cohort transcriptomic strategy. Differential expression analysis and weighted gene co-expression network analysis were used to define KBD-related gene sets in peripheral blood. Multiple feature-selection methods and machine-learning models were systematically compared to identify an optimal diagnostic panel. Model performance was evaluated across independent cohorts. SHAP analysis, transcription factor regulatory analysis, and immune cell deconvolution were applied to provide biological context for the selected genes. This approach was designed to establish a reproducible blood biomarker panel and to support further investigation of systemic immune and metabolic alterations in KBD.

## 2 Materials and methods

### 2.1 Data sources

Public transcriptomic datasets were retrieved from the Gene Expression Omnibus (GEO) and ArrayExpress databases using the keywords “Kashin–Beck disease”, “blood”, “PBMC”, and “cartilage”. Datasets were included if they met the following criteria: (i) samples were derived from patients with Kashin–Beck disease and non-KBD controls; (ii) transcriptomic profiling was performed using either RNA-seq or microarray platforms; and (iii) processed expression matrices and corresponding sample annotations were publicly available. Datasets lacking group labels or containing duplicate samples were excluded. When multiple samples from the same individual were present, only one sample was retained.

The final analysis included a whole-blood RNA-seq discovery cohort (GSE186593), a peripheral blood microarray training cohort (GSE59446), a PBMC microarray validation cohort (GSE32127), and an articular cartilage microarray validation cohort (E-MEXP-3196). A summary of all datasets is provided in **Table 1**.

**Table 1:** Summary of transcriptomic datasets included in this study.

Dataset	Platform	Experimental type	Tissue	Sample size (KBD/Control)
GSE186593	GPL16791	RNA-seq	Whole blood	5/5
GSE59446	GPL17897	Microarray	PBMCs	100/100
GSE32127	GPL7264	Microarray	PBMCs	4/4
E-MEXP-3196	A-MEXP-703	Microarray	Articular cartilage	4/4

Patients with Kashin–Beck disease met local diagnostic criteria. Healthy controls were matched by age and region. Individuals with rheumatoid arthritis, osteoarthritis, other major joint disorders, systemic inflammatory diseases, or malignancies were excluded.

### 2.2 Dataset preprocessing and normalization

All datasets were processed separately according to platform-specific workflows, and no direct cross-platform normalization was performed. Instead, each dataset was normalized within platform, and downstream analyses were conducted using dataset-specific expression matrices.

For the RNA-seq dataset (GSE186593), raw count matrices were obtained from GEO and annotated using the corresponding GRCh38 gene annotation file. Gene identifiers were mapped to gene symbols, and duplicate symbols were resolved by assigning unique identifiers. Low-abundance genes were filtered using the filterByExpr function in the edgeR (18) package. Genes with very low total counts were further excluded prior to downstream analysis. Expression data were then used for differential expression analysis with DESeq2 (19), and log-scale transformed expression values were generated where appropriate for visualization and downstream analyses.

For the microarray training cohort GSE59446, raw expression tables for Kashin–Beck disease and control samples were downloaded separately and merged by probe identifier to generate a unified expression matrix. Expression values were transformed using log_2_(x + 1) prior to downstream model training. Sample metadata were matched to the expression matrix by sample identifier to ensure consistent ordering. For the two-color microarray validation cohort GSE32127 and E-MEXP-3196, raw GPR files were processed using the limma (20) package. Red and green foreground and background intensities were extracted, followed by background correction using the normexp method. Corrected intensities were log_2_-transformed, and quantile normalization was applied across channels. Probe identifiers were mapped to gene symbols using the corresponding GPL annotation file. Probes without valid gene annotation were removed, and when multiple probes mapped to the same gene, probe-level expression values were aggregated to the gene level using the median.

To ensure consistency across datasets, all expression data were converted to gene-level matrices prior to downstream analysis. Because RNA-seq and microarray platforms differ in dynamic range and signal distribution, all modeling and validation procedures were performed within each dataset separately.

### 2.3 Differential expression analysis

Differential expression analysis was performed exclusively in the RNA-seq discovery cohort (GSE186593) to identify candidate genes associated with Kashin–Beck disease. No differential expression analysis was conducted in the microarray cohorts, which were instead used for model training and independent validation.

Raw count data were annotated using the corresponding GRCh38 gene annotation file, and Ensembl identifiers were mapped to gene symbols. Duplicate gene symbols were resolved by assigning unique identifiers. Low-abundance genes were filtered using the filterByExpr function in the edgeR package based on disease group. In addition, genes with very low total counts (row sum ≤ 15) were excluded prior to model fitting.

Differential expression analysis was performed using the DESeq2 package. An initial design formula including sex and group was specified. To account for unmeasured technical and biological heterogeneity, surrogate variables were estimated using the sva package (21) based on variance-stabilized expression data generated by the vst function. The final model included sex, surrogate variables, and disease group. P values were adjusted using the Benjamini–Hochberg method. Genes with an adjusted P value *<* 0.05 and |log_2_(FC)| ≥ 1.5 were considered differentially expressed.

### 2.4 Weighted gene co-expression network analysis

WGCNA (22) was performed using variance-stabilized expression data derived from the RNA-seq discovery cohort (GSE186593). The input matrix was obtained from the vst-transformed expression values generated by DESeq2. To reduce noise and computational burden, the top 10,000 genes ranked by variance were selected for network construction. Data quality was assessed using the goodSamplesGenes function to remove low-quality samples and genes. A soft-thresholding power was selected using the pickSoftThreshold function to approximate scale-free topology. An adjacency matrix was constructed and subsequently transformed into a topological overlap matrix (TOM). Hierarchical clustering was then performed to identify gene modules. Module eigengenes were calculated and correlated with disease status. Genes from modules significantly associated with Kashin–Beck disease were intersected with differentially expressed genes to define a co-expression-supported candidate gene set.

### 2.5 Functional enrichment analysis

The input gene set consisted of candidate genes identified from the intersection of differentially expressed genes and WGCNA module genes. Functional enrichment analysis was performed using the clusterProfiler (23) package in R. Gene Ontology (GO) and KEGG pathway enrichment analyses were conducted. P values were adjusted for multiple testing using the Benjamini–Hochberg method. Enriched terms with adjusted P value *<* 0.05 were considered statistically significant. Heatmaps were generated using the Bioinformatics online analysis platform (https://www.bioinformatics.com.cn).

### 2.6 Feature selection and machine learning model construction

For machine-learning analysis, only genes shared between the training and validation datasets were retained. The expression matrices were converted to sample-by-feature format before model fitting. The training cohort was centered and scaled, and the validation cohorts were standardized separately according to cohort labels using the same predefined scaling procedure. A fixed random seed (“set.seed(777)”) was used during feature screening and model training to improve reproducibility. Hyperparameters were selected using algorithm-specific internal cross-validation procedures or predefined settings according to the corresponding model class.

A multi-strategy machine learning framework was implemented to reduce method-specific bias. Feature selection methods included LASSO, ridge regression, elastic net with different *α* values, stepwise logistic regression, glmBoost (24, 25), and random forest (26). Classification algorithms included logistic regression, support vector machine (SVM), linear discriminant analysis (LDA), partial least squares logistic regression (plsRglm), random forest (26), gradient boosting machine (GBM), XGBoost (27), glmBoost (24), and Naive Bayes. These feature selection methods and classifiers were combined to construct multiple modeling pipelines. Model parameters were determined using cross-validation. Regularization parameters for elastic net models were selected using 10-fold cross-validation. Boosting-based models and tree-based models were tuned using cross-validation to determine optimal model complexity. Random forest models were trained with 1,000 trees. Additional implementation details are provided in the GitHub repository.

Models were trained in the GSE59446 cohort and evaluated using receiver operating characteristic (ROC) analysis. To reduce overfitting and model selection bias, trained models were applied to independent external cohorts without retraining. Model selection was based on mean AUC across validation cohorts rather than training performance.

For the final selected model, AUC with 95% confidence intervals (CIs) was estimated using bootstrap resampling. Sensitivity and specificity were calculated at the optimal cutoff determined by the Youden index.

### 2.7 Decision curve analysis

Decision curve analysis (DCA) (28) was performed to evaluate the potential clinical utility of the four-gene model. Net benefit was calculated across a range of threshold probabilities and compared with two default strategies: classifying all individuals as having Kashin–Beck disease (treat-all) and classifying none as having the disease (treat-none). DCA was implemented using the rmda package in R. The analysis was conducted separately in the training and validation cohorts based on the predicted risk scores generated by the final model. Threshold probabilities ranging from 0 to 1 were evaluated, and net benefit curves were used to assess the clinical usefulness of the model under different decision thresholds.

### 2.8 SHAP-based model interpretability

Shapley additive explanations (SHAP) (29) were applied to assess gene-level contributions in the final four-gene model (*C4B*, *AQP1*, *HBA2*, and *ACSL6*). The model was trained in the GSE59446 cohort using Z-score–standardized expression values. SHAP values were calculated on the logit scale to quantify the marginal contribution of each gene to the predicted risk score. Global feature importance was summarized using mean absolute SHAP values. A SHAP summary plot was generated to visualize the relationship between gene expression levels and prediction impact.

### 2.9 Gene set enrichment analysis based on model-derived risk score

Gene set enrichment analysis (GSEA) (30) was performed in the discovery cohort to explore pathways associated with the diagnostic model. Gene expression values were transformed as log_2_(count + 1). The trained multigene model was used to calculate a continuous transcriptomic risk score for each sample. Samples were stratified into high- and low-risk groups according to the median score. Differential expression between groups was assessed using limma (20), and moderated t-statistics were used to rank genes for GSEA. Hallmark and KEGG MEDICUS gene sets were analyzed using clusterProfiler (23). Normalized enrichment scores and Benjamini–Hochberg adjusted P values were used to identify significant pathways.

### 2.10 Real-time quantitative PCR

Peripheral blood samples (4 mL) were collected from human participants into EDTA-K2 anticoagulation tubes. Total RNA was extracted using TRIzol reagent and quantified using a NanoDrop spectrophotometer. Complementary DNA (cDNA) was synthesized using HiScript II Q RT SuperMix. Quantitative PCR was performed using gene-specific primers and Taq Pro Universal SYBR qPCR Master Mix. Gene expression levels were normalized to GAPDH. Clinical characteristics of the qPCR validation cohort and primer sequences are provided in **Supplementary Table 1**.

The studies involving human participants were approved by the Institutional Review Board of Shaanxi Provincial People’s Hospital (Approval No. 2023K-S138). All procedures were conducted in accordance with the Declaration of Helsinki. Written informed consent was obtained from all participants or their legal guardians prior to sample collection.

### 2.11 Immune cell infiltration analysis

Immune cell composition in peripheral blood was estimated using the CIBERSORT algorithm (31) with the LM22 signature matrix. For the GSE186593 cohort, raw count data were transformed as log_2_(count + 1). Expression profiles were intersected with LM22 signature genes. CIBERSORT was run with 100 permutations and quantile normalization disabled. Relative proportions of 22 immune cell types were estimated. Group differences were assessed using the Wilcoxon rank-sum test. Spearman correlation coefficients were calculated between hub-gene expression and immune cell fractions. P values *<* 0.05 were considered significant.

### 2.12 Transcription factor regulatory network analysis

Transcription factor regulatory analysis was performed to identify upstream regulators of the hub genes. The TRRUST v2 (32) and RegNetwork (33) databases were queried for transcription factors with documented regulatory evidence and confidence scores greater than 0.9. Differentially expressed transcription factors were retained. Pearson correlation coefficients were calculated between transcription factors and hub genes.

### 2.13 Genetic support from blood cis-eQTL and PheWAS

Blood cis-expression quantitative trait loci (cis-eQTLs) for the four diagnostic genes were queried using the GTEx v8 (34) Whole Blood dataset. For each gene, the lead cis-eQTL was defined as the variant with the strongest association signal. Phenome-wide association analyses (PheWAS) were performed for each lead cis-eQTL using the CTG Lab PheWAS Atlas (35). Traits reaching platform-defined significance thresholds were extracted and categorized by phenotype domain. PheWAS results were visualized as Manhattan-style plots and combined into a multi-panel figure. Formal colocalization analysis was not conducted due to the absence of publicly available genome-wide KBD GWAS summary statistics.

### 2.14 Statistical analysis

All statistical analyses were performed in R unless otherwise specified. Continuous variables are presented as mean ± standard deviation or median with interquartile range. Group comparisons were conducted using Student’s t-test or the Mann–Whitney U test. Categorical variables were analyzed using the chi-square test or Fisher’s exact test. Multiple testing was controlled using the Benjamini–Hochberg method. Correlations were assessed using Pearson or biweight midcorrelation coefficients. A two-sided P value *<* 0.05 was considered statistically significant. Calibration analysis was performed to assess the agreement between predicted probabilities and observed outcomes. Calibration performance was evaluated using calibration plots, Brier scores, calibration intercepts, and calibration slopes.

## 3 Results

### 3.1 Identification of KBD-related blood gene sets by differential expression and WGCNA

**Figure 1:**
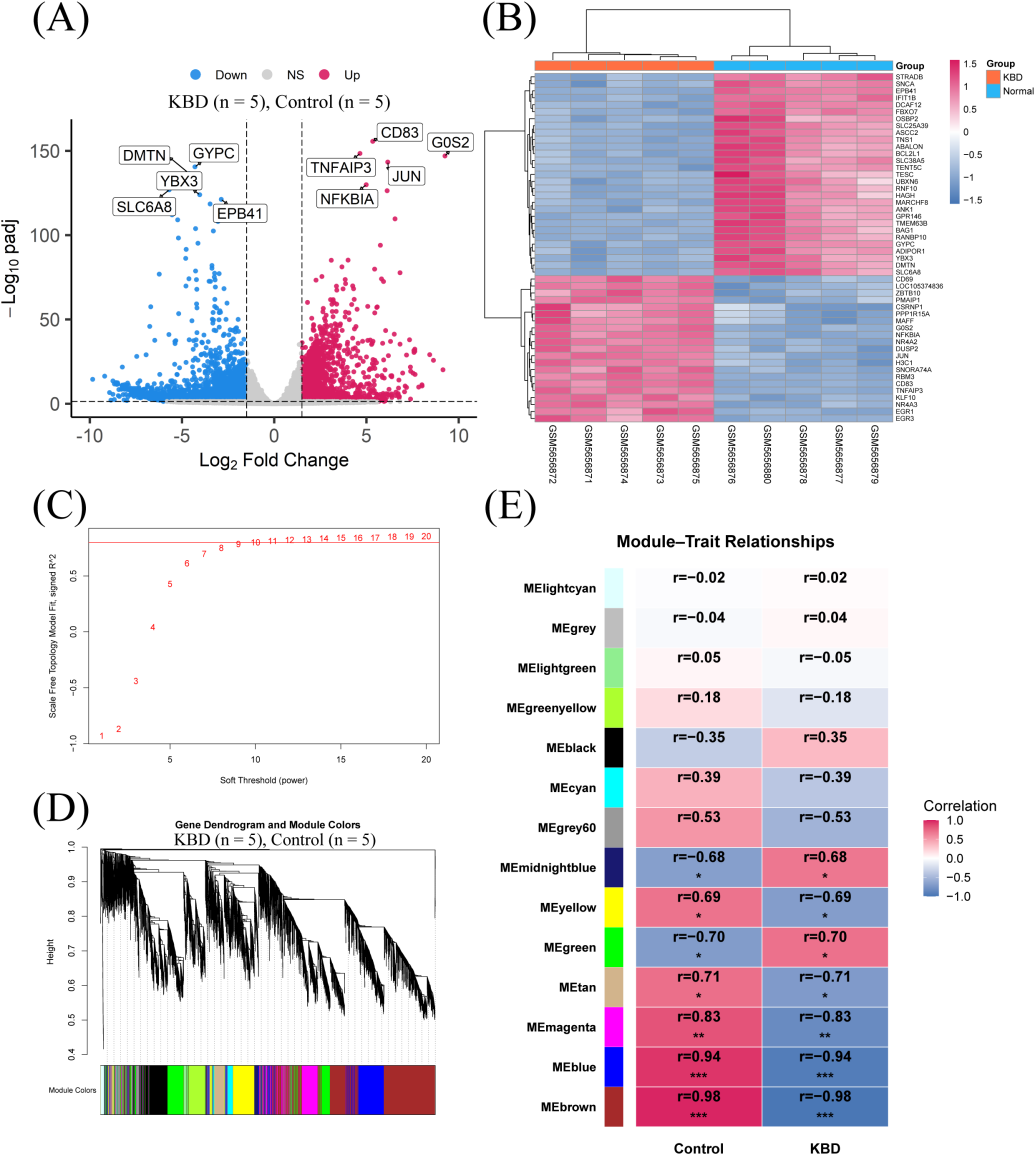
Differential expression analysis and weighted gene co-expression network analysis in the discovery cohort. **(A)** Volcano plot showing differentially expressed genes between KBD and control samples, with representative upregulated and downregulated genes labeled. **(B)** Heatmap of the top 50 differentially expressed genes, illustrating clear separation between KBD and control samples. **(C)** Scalefree topology model fit index across different soft-thresholding powers used to select the optimal parameter for network construction. **(D)** Gene dendrogram and module colors generated by hierarchical clustering based on the topological overlap matrix. **(E)** Module–trait relationship heatmap showing correlations between module eigengenes and clinical status (KBD vs. control).

Differential expression analysis was performed in the peripheral blood transcriptome cohort GSE186593 (5 KBD, 5 controls). DESeq2 detected 23,416 transcripts in total. Using adjusted P values *<* 0.05 and $|\log_{2}\left(\text{FC}\right)|\geq 1.5$ as thresholds, 1,839 genes were upregulated and 2,913 genes were downregulated in KBD compared with controls (**Figure 1A**). A heatmap of the top 50 differentially expressed genes showed clear separation between KBD and control samples, with the two groups forming distinct branches in the clustering tree (**Figure 1B**).

To identify coordinated expression patterns, weighted gene co-expression network analysis was conducted using the 10,000 most variable genes. Soft-thresholding analysis identified an appropriate power that achieved a scale-free topology fit for network construction (**Figure 1C**). Genes were clustered based on the topological overlap matrix, and multiple co-expression modules were detected, each represented by a distinct color (**Figure 1D**). Correlation analysis between module eigengenes and clinical status showed that several modules were strongly associated with KBD. The brown and blue modules displayed strong negative correlations with KBD (|*r*| approaching 1, *p <* 0.001), whereas the green and midnightblue modules showed moderate positive correlations (|*r*| around 0.7, *p <* 0.05) (**Figure 1E**). The brown module was selected for downstream analyses based on its strong association with KBD status. Specifically, this module showed the highest module–trait correlation and the highest module significance among all identified modules. Module significance was defined as the average gene significance of genes within the module.

### 3.2 Functional enrichment of KBD-related candidate genes

**Figure 2:**
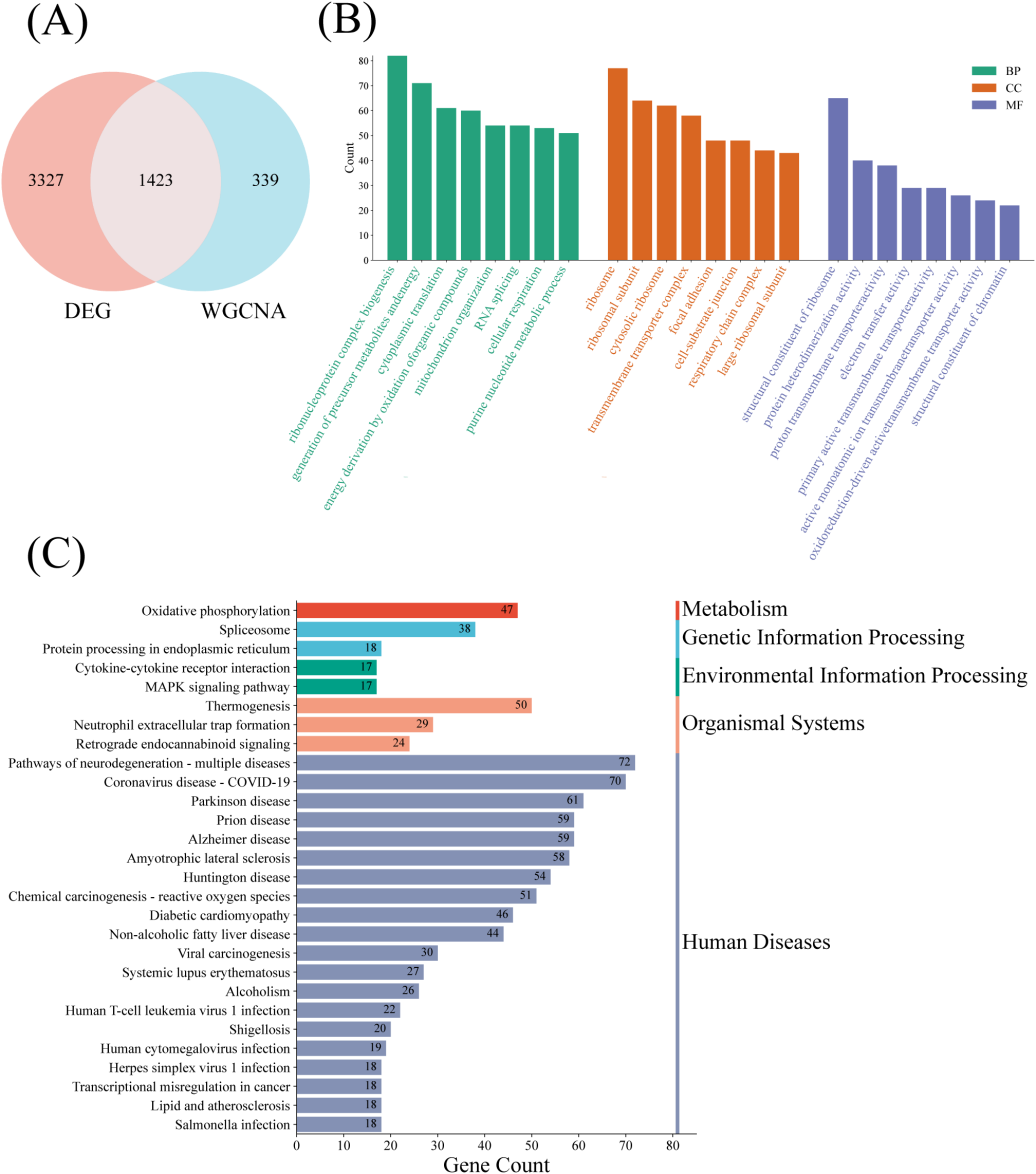
Functional enrichment analysis of KBD-related candidate genes. **(A)** Venn diagram showing the overlap between differentially expressed genes and genes from the KBD-associated WGCNA module, yielding 1,423 candidate genes. **(B)** Gene Ontology enrichment results for biological process (BP), cellular component (CC), and molecular function (MF) categories. **(C)** KEGG pathway enrichment analysis of the candidate gene set, highlighting significantly enriched pathways across metabolism, genetic and environmental information processing, organismal systems, and human disease categories.

A total of 1,423 candidate genes were obtained by intersecting significant DEGs with the brown module (**Figure 2A**). Gene Ontology enrichment analysis showed significant enrichment of biological processes involved in RNA processing, cytoplasmic translation, mitochondrial organization, and cellular respiration (**Figure 2B**). Enriched cellular components included ribosomal structures, respiratory chain complexes, and transmembrane transporter complexes. Enriched molecular functions involved structural constituents of ribosomes and multiple oxidoreductase- and transporter-related activities. KEGG pathway analysis showed predominant enrichment in pathways annotated under the Human Diseases category, particularly neurodegenerative and immune-related disorders. Additional enrichment was observed in pathways involved in mitochondrial metabolism, RNA processing, cytokine signaling, and MAPK signaling (**Figure 2C**).

Overall, GO and KEGG enrichment analyses revealed convergent involvement of mitochondrial metabolism, RNA processing, immune signaling, and stress-response pathways within the candidate gene set. These pathways provided a functional framework for subsequent analyses.

### 3.3 Construction and validation of a blood-based machine learning diagnostic model for KBD

**Figure 3:**
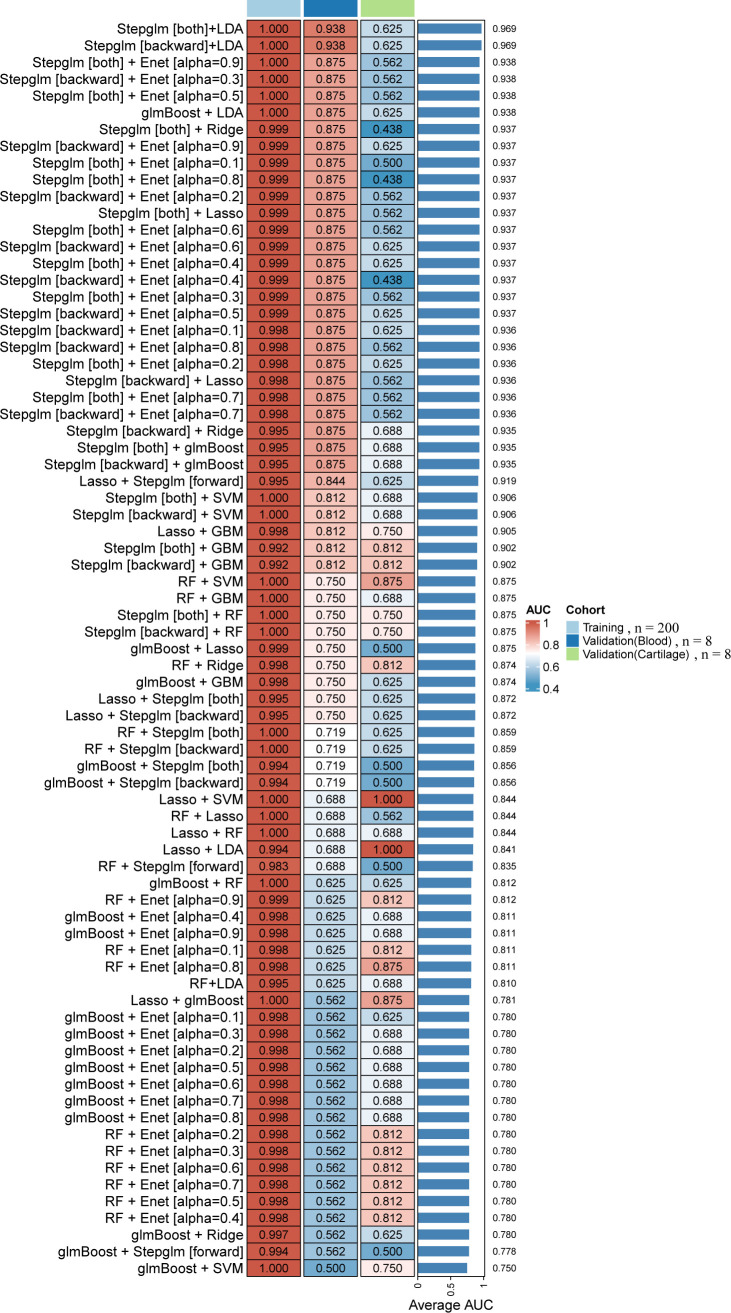
Performance comparison of machine-learning pipelines for KBD diagnosis across multiple cohorts. Heatmap of AUC values for 113 combinations of feature-selection and classification algorithms evaluated in the training cohort, a blood validation cohort, and a cartilage validation cohort. Rows represent individual pipelines and columns represent cohorts. The bar plot indicates the mean AUC across cohorts. The Stepglm[both] + LDA model showed the highest mean AUC and stable performance.

The peripheral blood microarray cohort GSE59446 (100 KBD and 100 controls) was used as the training set. After intersecting the expression matrix with the candidate gene list, 27 genes were retained for model development (**Figure 3**). A total of 113 classification models were constructed using different combinations of feature-selection and machine-learning algorithms. Model performance was evaluated using cross-validation in the training set and further assessed in independent external cohorts.

Most models achieved cross-validated AUC values above 0.90 in the training set. In the independent blood validation cohort GSE32127, the average AUC across models was approximately 0.81, whereas performance was lower in the cartilage cohort E-MEXP-3196 (average AUC 0.65). The mean AUC across datasets ranged from 0.75 to 0.97. Among all pipelines, the Stepglm[both] + LDA model showed the most stable performance, with a mean AUC of 0.969 (**Figure 3**). This model selected four genes—*C4B*, *AQP1*, *HBA2*, and *ACSL6*—as the optimal diagnostic feature set.

To further evaluate model performance, AUC values with 95% confidence intervals (CIs) were calculated using bootstrap resampling. In the training cohort (GSE59446), the four-gene model achieved an AUC of 1.000 (95% CI, 0.999–1.000). In the external validation cohorts, the AUC was 0.938 (95% CI, 0.764–1.000) in GSE32127 and 0.625 (95% CI, 0.135–1.000) in E-MEXP-3196. Sensitivity and specificity were calculated at the optimal cutoff determined by the Youden index for each cohort. The model showed consistently high sensitivity across datasets, while specificity varied between cohorts, particularly in the smaller validation dataset (**Supplementary Table 1**).

Decision curve analysis was performed to assess the potential clinical utility of the four-gene model. In the training cohort, the model showed a higher net benefit than the treat-all and treat-none strategies across a broad range of threshold probabilities. In the validation cohort, a net benefit was observed at lower threshold probabilities, although this advantage decreased at higher thresholds. These results suggest that the clinical utility of the model may depend on the selected decision threshold (**Supplementary Figure 1**).

To assess whether model performance exceeded that expected by chance, a random-gene benchmarking analysis was performed. The four-gene signature consistently outperformed randomly generated gene sets, indicating that its performance was unlikely to arise from random feature selection (**Supplementary Figure 2**). Calibration analysis was performed to further evaluate model performance. In the training cohort, the model showed acceptable agreement between predicted probabilities and observed outcomes. In the external validation cohort, calibration was less stable, likely owing to the limited sample size of the available validation data. Therefore, calibration results in the validation cohorts should be interpreted cautiously and regarded as exploratory (**Supplementary Figure 3**).

**Figure 4:**
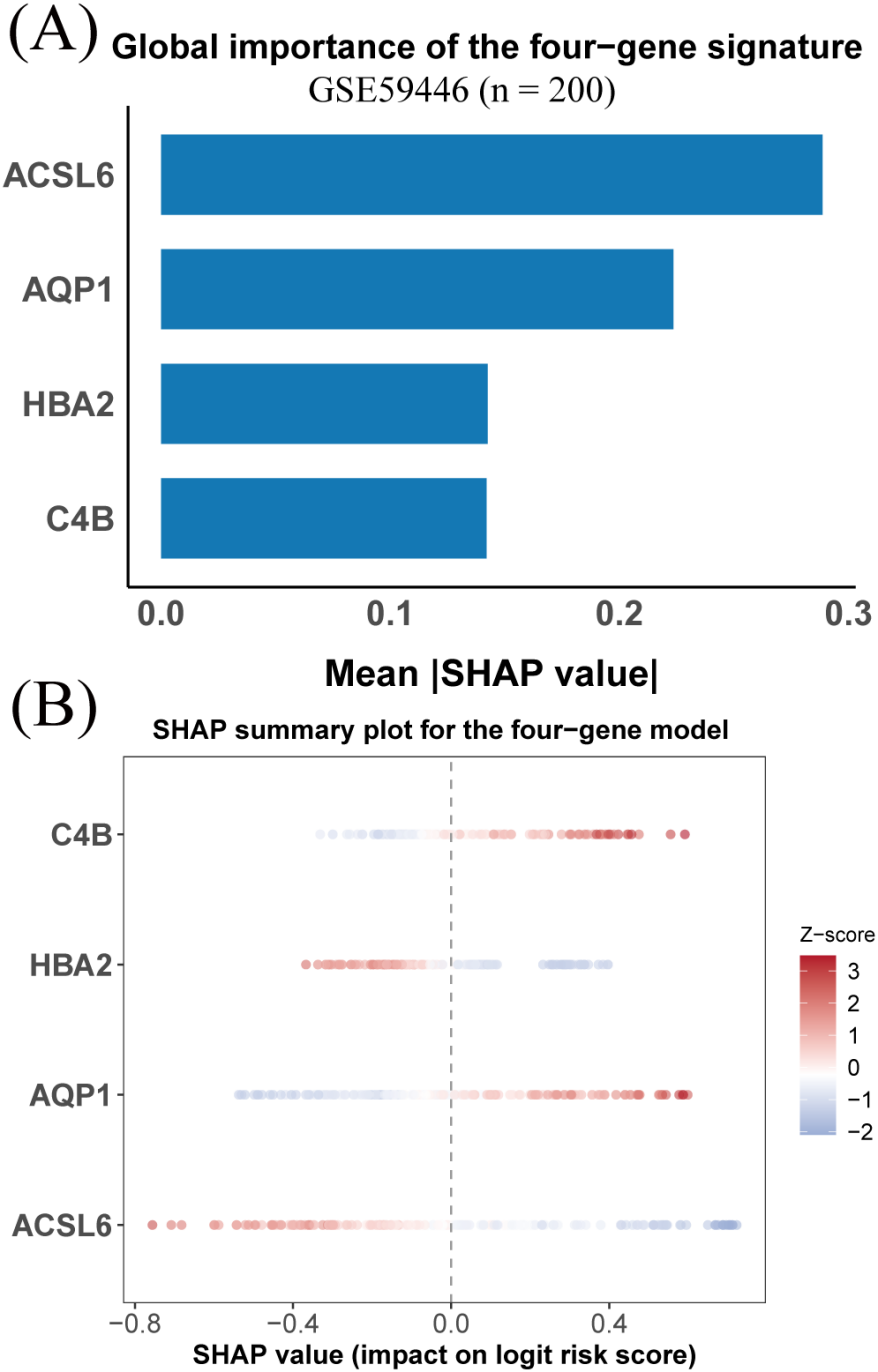
SHAP-based interpretation of the four-gene diagnostic model. **(A)** Mean absolute SHAP values indicating the relative contribution of each gene. **(B)** SHAP summary plot showing the relationship between standardized gene expression levels and their effects on the predicted risk score.

SHAP analysis was applied to the optimal Stepglm[both] + LDA model to quantify gene-level contributions. *ACSL6* and *AQP1* showed the highest mean absolute SHAP values, followed by *HBA2* and *C4B* (**Figure 4A**). The SHAP summary plot demonstrated consistent relationships between gene expression levels and their contributions to the predicted risk score, with more extreme expression values associated with larger SHAP effects (**Figure 4B**).

### 3.4 Single-gene performance and pathway characteristics of the diagnostic panel

**Figure 5:**
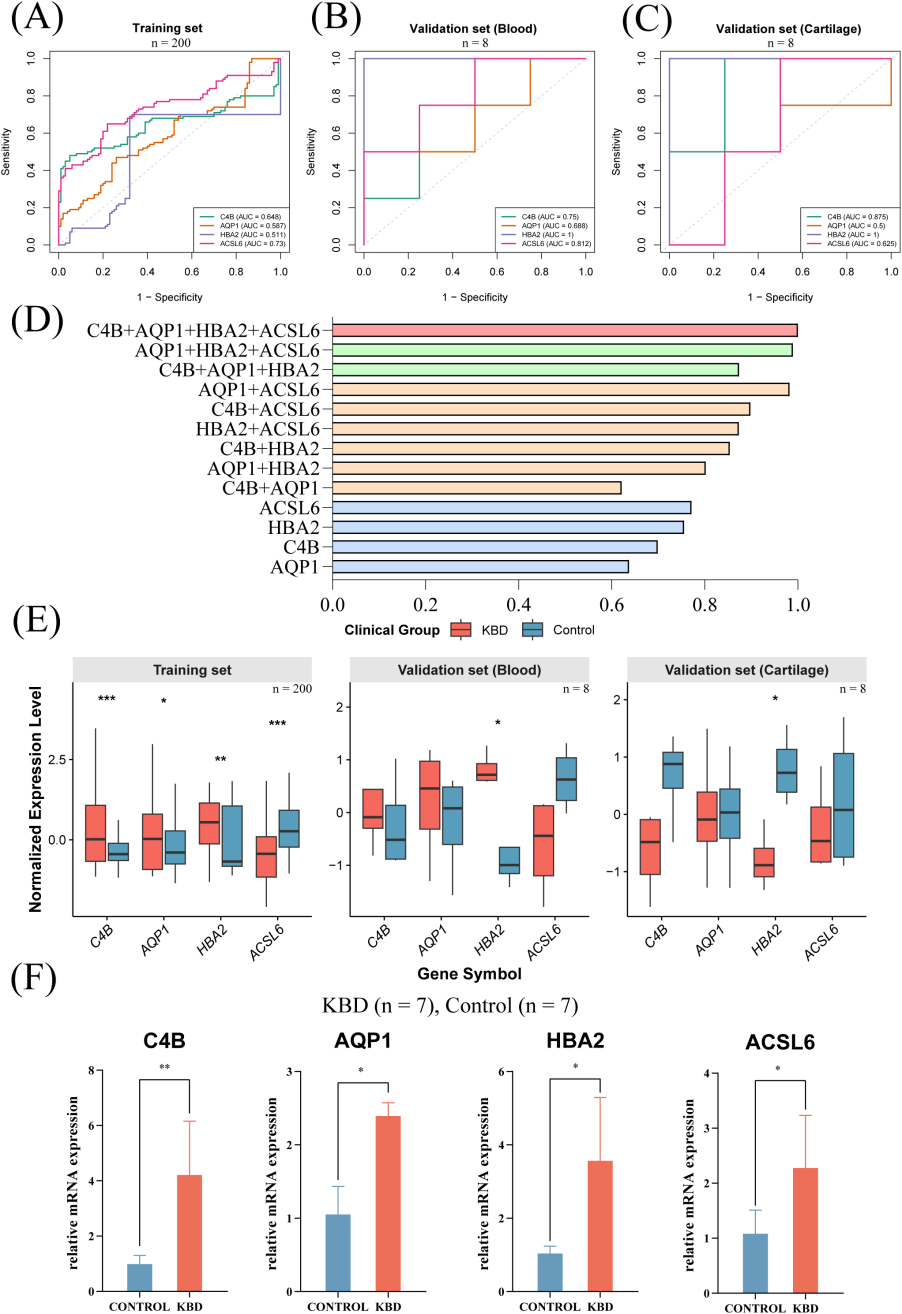
Single-gene and multi-gene diagnostic performance and validation of the four-gene panel. **(A–C)** ROC curves across cohorts. **(D)** AUC distributions of gene combinations. **(E)** Gene expression differences between KBD and controls. **(F)** qPCR validation in an independent cohort.

The discriminative performance of each gene was evaluated individually (**Figures 5A–C**). In the training cohort (GSE59446), single-gene AUC values ranged from 0.511 to 0.730, with *ACSL6* showing the highest AUC (0.730), followed by *C4B* (0.648), *AQP1* (0.587), and *HBA2* (0.511) (**Figure 5A**). In the blood validation cohort (GSE32127), *HBA2* achieved an AUC of 1.000, whereas *ACSL6*, *C4B*, and *AQP1* achieved AUCs of 0.812, 0.750, and 0.688, respectively (**Figure 5B**). In the cartilage validation cohort (E-MEXP-3196), *HBA2* and *C4B* showed AUCs of 1.000 and 0.875, while *ACSL6* reached 0.625 and *AQP1* showed no discriminative ability (AUC = 0.500) (**Figure 5C**).

To assess the additive value of multi-gene integration, all one-, two-, three-, and four-gene combinations were evaluated in the training cohort (**Figure 5D**). The full four-gene combination (*C4B*+*AQP1*+*HBA2*+*ACSL6*) achieved the highest AUC, and the best three-gene combinations also outperformed the corresponding single-gene predictors, supporting a synergistic diagnostic effect of the panel(**Figure 5D**).

Across all cohorts, consistent case–control expression trends were observed for the four diagnostic genes (**Figure 5E**). In the training cohort, all four genes were significantly differentially expressed between KBD patients and controls. In the external blood validation cohort, only *HBA2* reached statistical significance, whereas the remaining genes showed concordant directions of change, likely reflecting limited sample size and platform-related differences. In the cartilage validation cohort, the four genes exhibited distinct expression patterns, suggesting tissue-specific regulatory features. Quantitative PCR validation in an independent cohort (7 KBD patients and 7 controls) confirmed significant differential expression of all four genes (all *P <* 0.05) (**Figure 5F**). The direction of change for *C4B*, *AQP1*, and *HBA2* was consistent with the transcriptomic analyses, whereas *ACSL6* showed a discordant direction, indicating potential sensitivity to assay platform and/or peripheral blood cell composition.

**Figure 6:**
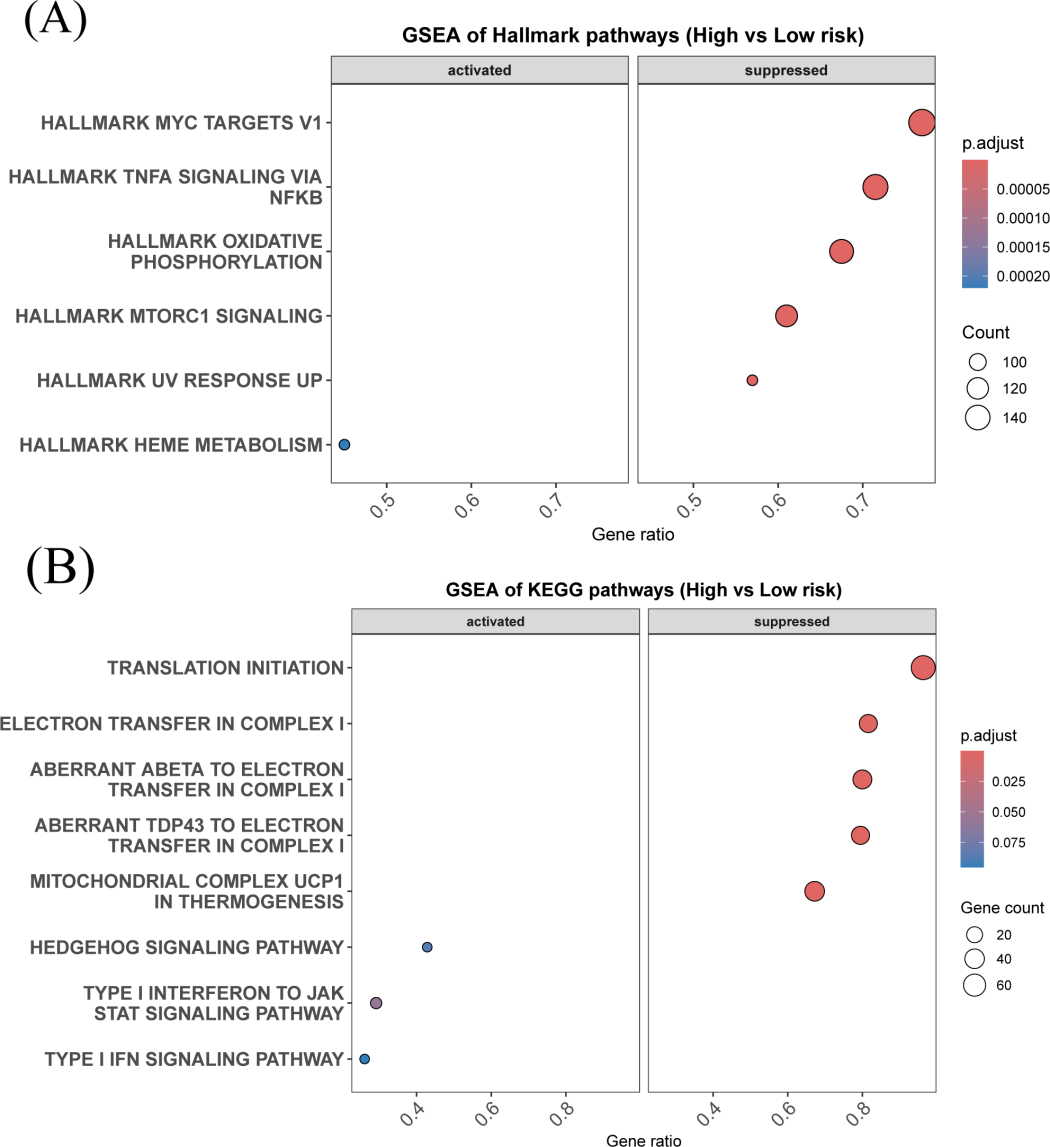
Gene set enrichment analysis based on the model-derived risk score in the discovery cohort (GSE186593). **(A)** GSEA of Hallmark gene sets comparing high-risk and low-risk groups, highlighting pathways related to immune signaling, metabolism, and cellular stress responses. **(B)** GSEA of KEGG MEDICUS gene sets comparing high-risk and low-risk groups, showing enrichment of pathways associated with translation initiation, mitochondrial electron transport, and immune-related signaling. Dot size represents the number of genes in each pathway, and color indicates the adjusted P value.

To place the diagnostic panel in a pathway context, gene set enrichment analysis was performed using the model-derived risk score. In Hallmark analysis, higher predicted KBD risk was associated with significant negative enrichment of several immune- and metabolism-related gene sets, including TNF-*α*/NF-*κ*B signaling, inflammatory response, and oxidative phosphorylation (**Figure 6A**), indicating reduced activity of classical inflammatory pathways and mitochondrial energy metabolism. In contrast, KEGG MEDICUS analysis revealed positive enrichment of interferon-related pathways in the high-risk group, alongside negative enrichment of translation initiation and multiple mitochondrial complex I and electron transfer modules (**Figure 6B**). Together, these results suggest a shift in immune signaling characterized by selective activation of interferon-related responses and suppression of broader inflammatory, translational, and metabolic programs.

### 3.5 Transcription factor regulatory networks of the KBD diagnostic signature

**Figure 7:**
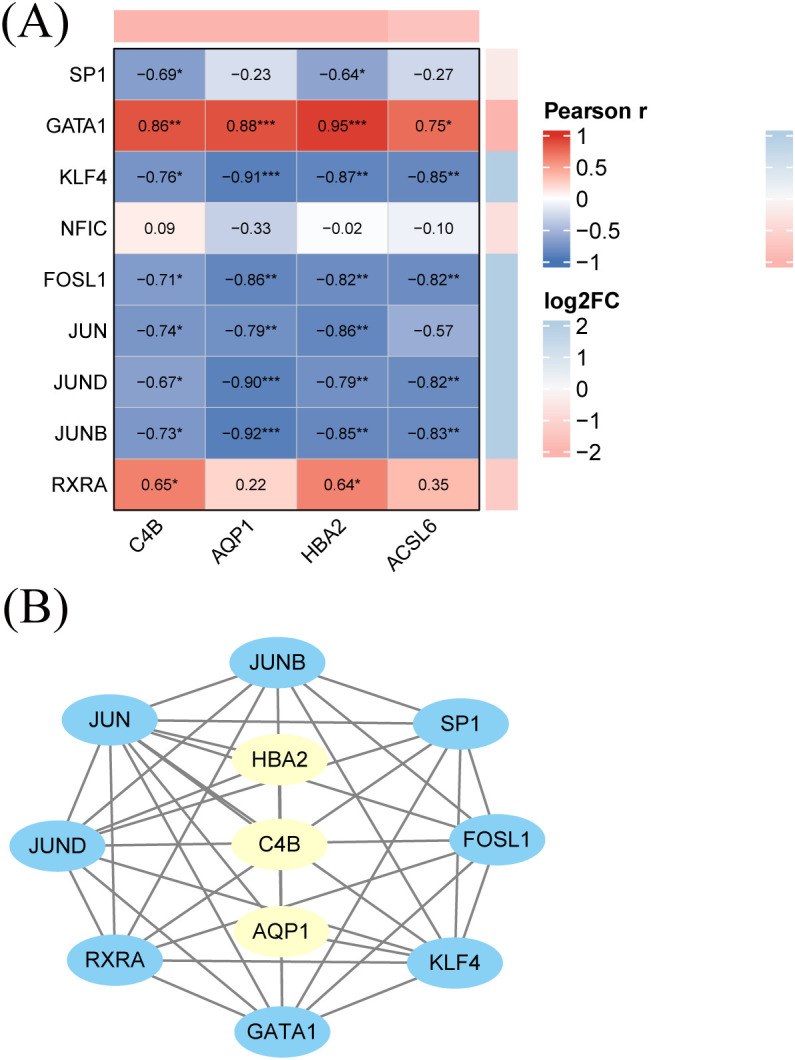
Transcription factor–hub gene associations and regulatory network of the KBD diagnostic signature. **(A)** Pearson correlation heatmap between selected transcription factors and the four hub genes (*C4B*, *AQP1*, *HBA2*, and *ACSL6*) in the discovery cohort, with log_2_ fold-change values shown alongside. **(B)** TF–hub gene regulatory network visualizing putative upstream regulators and their connections with the four hub genes.

A total of 20 transcription factors (TFs) were identified from the TRRUST v2 and RegNetwork databases as putative upstream regulators of *C4B*, *AQP1*, *HBA2*, and *ACSL6*. Among them, nine TFs showed significant differential expression in the discovery cohort (**Table 2**). Their expression levels were extracted from the GSE186593 RNA-seq dataset, and Pearson correlation coefficients between each TF and the four hub genes were calculated (**Figure 7A**). Log_2_ fold-change values are displayed alongside the correlation heatmap.

**Table 2:** Differentially expressed transcription factors predicted to regulate the four diagnostic genes in the discovery cohort.

TF	log_2_(FC)	*P* value	adjusted *P* value	Target gene
SP1	-0.4250	1.48×10^−2^	3.63×10^−2^	*C4B*
GATA1	-3.2590	1.26×10^−46^	2.34×10^−44^	*HBA2*
KLF4	2.2162	2.72×10^−33^	2.30×10^−31^	*HBA2*
NFIC	-0.7179	7.50×10^−5^	3.38×10^−4^	*HBA2*
FOSL1	4.7951	7.37×10^−40^	9.70×10^−38^	*AQP1*
JUN	6.1585	8.32×10−148	4.87×10−144	*AQP1*
JUND	2.1502	1.83×10^−29^	1.16×10^−27^	*AQP1*
JUNB	2.7535	4.59×10^−45^	7.73×10^−43^	*AQP1*
RXRA	-1.3287	1.13×10^−8^	1.03×10^−7^	*ACSL6*

Most TF–gene pairs showed significant correlations. *GATA1* showed strong positive correlations with all four genes (*r* = 0.75–0.95, *p <* 0.01). *RXRA* was positively correlated with *C4B* and *HBA2* (*r* ≈ 0.65, *p <* 0.05). In contrast, *KLF4* and AP-1 family members (*FOSL1*, *JUN*, *JUND*, and *JUNB*) showed negative correlations with the four genes, with most correlation coefficients below −0.7 (*p <* 0.05). Together, these findings define a focused TF–gene regulatory landscape associated with the diagnostic signature.

### 3.6 Altered immune cell composition and hub gene–immune associations in KBD

**Figure 8:**
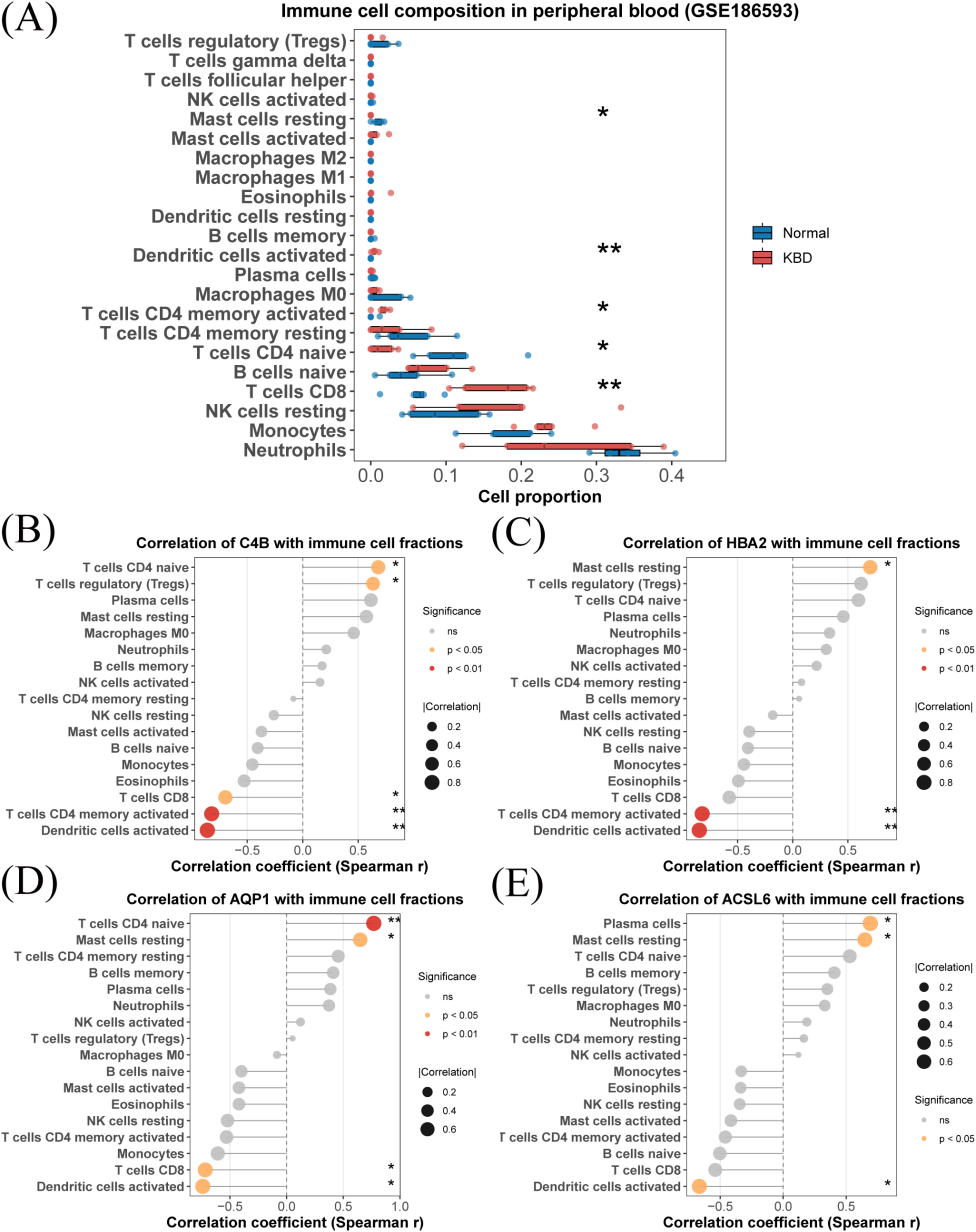
Immune cell composition and hub gene–immune associations in the discovery cohort (GSE186593). **(A)** Comparison of immune cell proportions between KBD and control samples estimated by CIBERSORT using the LM22 signature matrix. **(B–E)** Spearman correlation analysis between inferred immune cell fractions and expression levels of *C4B*, *HBA2*, *AQP1*, and *ACSL6*, respectively. Dot size represents the absolute value of the correlation coefficient, and color indicates statistical significance.

Immune cell proportions were estimated using CIBERSORT with the LM22 signature matrix in the discovery cohort. Several immune cell types differed significantly between KBD and control samples, including neutrophils, monocytes, resting NK cells, CD8^+^ T cells, and naive B cells (Wilcoxon test, *p <* 0.05) (**Figure 8A**).

Associations between hub-gene expression and inferred immune cell fractions were further examined. All four hub genes showed significant correlations with multiple immune cell subsets, with most absolute Spearman correlation coefficients exceeding |*r*| = 0.5 (**Figures 8B–E**). *AQP1* was correlated with activated dendritic cells, CD8^+^ T cells, monocytes, and memory CD4^+^ T-cell subsets. *C4B* was mainly associated with activated dendritic cells and memory CD4^+^ T cells. *HBA2* was associated with activated dendritic cells and CD8^+^ T cells, whereas *ACSL6* correlated with activated dendritic cells, CD8^+^ T cells, and naive B cells.

### 3.7 Genetic support for the diagnostic genes from blood cis-eQTL and PheWAS

All four diagnostic genes showed significant cis-eQTL signals in GTEx Whole Blood, indicating that their expression levels are under genetic regulation in peripheral blood. The lead cis-eQTL variants were rs389884 for *C4B*, rs73685792 for *AQP1*, rs13335497 for *HBA2*, and rs257389 for *ACSL6*. In contrast, previously reported KBD-associated variants did not show detectable cis-eQTL effects in GTEx Whole Blood.

**Figure 9:**
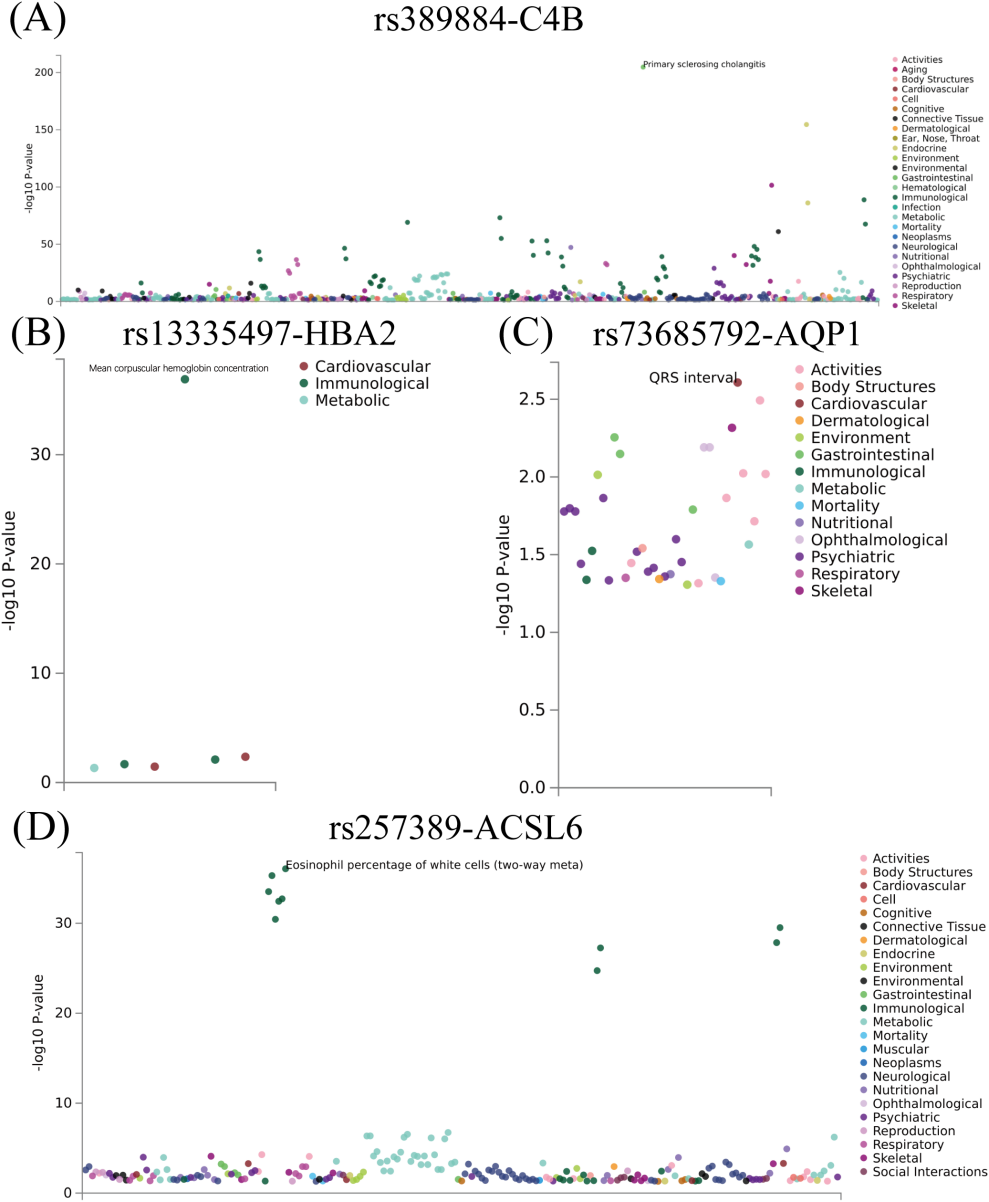
Phenome-wide association analyses of lead blood cis-eQTL variants for the four diagnostic genes. **(A)** PheWAS plot for rs389884, the cis-eQTL variant of *C4B*. **(B)** PheWAS plot for rs13335497, the cis-eQTL variant of *HBA2*. **(C)** PheWAS plot for rs73685792, the cis-eQTL variant of *AQP1*. **(D)** PheWAS plot for rs257389, the cis-eQTL variant of *ACSL6*. The x-axis represents phenotype categories, and the y-axis shows the –log_10_(P) values for variant–trait associations.

PheWAS analyses of the four lead cis-eQTL variants revealed associations across multiple phenotype domains (**Figure 9**). The associated traits were predominantly enriched in immune-related phenotypes, hematological traits, and metabolic or cardiometabolic measures. Several variants were also associated with inflammatory and musculoskeletal-related traits. Although these phenotypes are not specific to KBD, their enrichment patterns are consistent with the immune dysregulation and metabolic alterations observed in the blood transcriptomic analyses, providing genetic context for the four-gene diagnostic panel.

## 4 Discussion

This study identified a four-gene blood-based signature associated with Kashin–Beck disease using a multi-cohort transcriptomic and machine-learning framework. The model showed discriminatory ability across independent blood cohorts and retained partial classification ability in cartilage tissue. These findings suggest that the identified gene panel may reflect both systemic and joint-related aspects of the disease. Additional analyses, including transcription factor inference, immune cell deconvolution, pathway enrichment, and genetic annotation, provided preliminary biological context for the signature.

Previous studies have shown that KBD involves not only cartilage degeneration and growth plate damage, but also broader disturbances in extracellular matrix homeostasis, apoptosis, oxidative stress, mitochondrial metabolism, and inflammatory regulation (15, 36). Blood-based studies have likewise reported altered immune-related signaling and systemic transcriptomic changes in patients with KBD (5, 37). In the present study, enrichment analyses consistently pointed to immune-related pathways, complement-associated processes, lipid metabolism, oxygen-related biology, and mitochondrial functions. Taken together, these findings support the view that peripheral blood may capture part of the systemic molecular context of KBD rather than merely reflecting nonspecific secondary changes.

The four genes in the final model are biologically coherent in this context. *C4B* is a component of the complement system and may reflect altered innate immune activation. *AQP1* is involved in water transport and vascular permeability and may be relevant to microcirculatory regulation and tissue fluid homeostasis. *HBA2* is related to oxygen transport and may reflect hypoxia-related or erythroid-associated alterations. *ACSL6* is involved in lipid metabolism and may be linked to membrane remodeling and metabolic adaptation. Although each gene alone is not specific to KBD, their combination is consistent with a broader pattern involving immune activity, vascular or oxygen-related processes, and metabolic dysregulation. This integrated pattern may be more informative than any single marker alone.

This interpretation is also supported by the downstream analyses. Risk score-based enrichment suggested that samples with higher predicted KBD risk showed altered activity in pathways related to inflammation, mitochondrial function, oxidative phosphorylation, and protein synthesis. Immune deconvolution further indicated differences in several immune cell subsets between KBD and controls, and the observed correlations between hub-gene expression and inferred immune fractions suggest that the panel may partly capture systemic immune remodeling. In addition, transcription factor analysis suggested a regulatory context involving factors linked to inflammatory and differentiation-related programs. Although these analyses do not establish mechanism, they support the biological plausibility of the selected genes and indicate that the signature is not a purely statistical construct.

The cross-tissue behavior of the model is also of interest. The four-gene panel was derived from blood-based discovery and training cohorts, yet it retained partial discriminatory ability in cartilage tissue. This does not imply that blood and cartilage share identical expression patterns. Rather, it suggests that at least part of the signal captured by the model may reflect disease-relevant processes that are present across tissue contexts. In KBD, where direct sampling of affected cartilage is difficult in living individuals, this feature supports further exploration of blood-based biomarkers, even if the biological correspondence between blood and cartilage remains indirect and incomplete.

At the same time, these biological interpretations should remain cautious. The current findings are based on transcriptomic association rather than direct functional validation. The signature should therefore be viewed as a compact transcriptomic correlate of disease status that may reflect immune, metabolic, and oxygen-related alterations in KBD, rather than as proof of a defined causal pathway.

RT-qPCR validation further supported differential expression of the four genes, although *ACSL6* showed an inconsistent direction compared with the transcriptomic datasets. This discrepancy may reflect limited sample size, differences in cell composition, or platform-related effects. Importantly, the model was developed as a multigene panel, and its performance depends on the combined expression pattern rather than a single marker. In this setting, panel-level results are likely to be more informative than the direction of any individual gene alone.

Genetic association data provided additional context for the identified genes. Several genes in the panel have been linked to skeletal or growth-related traits in population studies (38–44). For example, *ACSL6* has been associated with human height, a trait relevant to KBD. These associations are not disease-specific, but they support the biological plausibility of the selected genes. Genetic analyses also showed that all four genes had detectable cis-eQTL signals in whole blood, suggesting that their expression is at least partly influenced by genetic variation. In contrast, previously reported KBD-associated variants did not show clear cis-eQTL effects in this dataset, which may reflect limited genetic data availability, insufficient statistical power, or tissue-specific regulation. Further studies with larger genetic datasets will be needed to clarify this point.

The present findings may have potential translational relevance because the four-gene panel is blood-based and minimally invasive. However, its clinical applicability remains uncertain at this stage and should be confirmed in larger independent cohorts.

Several limitations should be acknowledged. First, the sample size of the available public datasets was limited, particularly in the RNA-seq discovery cohort and the external validation cohorts. We systematically searched publicly available KBD-related transcriptomic datasets; however, because KBD is a geographically restricted endemic disorder, the number of available public cohorts remains very limited. As a result, the current external validation should be regarded as preliminary. The relatively small sample size reduces the precision of performance estimates and likely contributes to the wide confidence intervals observed in validation. It may also increase the risk of overfitting and model selection bias despite the use of cohort separation, internal cross-validation, external validation without retraining, and random-gene benchmarking. Therefore, larger independent and prospective cohorts are required to obtain more stable estimates of discrimination, calibration, and clinical utility. Second, the included datasets were generated from different platforms and experimental protocols, which introduces heterogeneity in signal distribution, dynamic range, and measurement characteristics. Although platform-specific preprocessing and independent validation were used to reduce these effects, residual technical variation cannot be completely excluded. Third, gene expression was analyzed at the bulk tissue level. Peripheral blood transcriptomic profiles reflect mixed cell populations, and observed differences may therefore partly arise from variation in cell composition rather than cell-intrinsic regulation alone. Although immune cell deconvolution was performed to provide additional context, this approach is reference-based and cannot replace direct cell-level measurement. Future studies using single-cell or spatial transcriptomic approaches will be needed to better resolve cell-type–specific effects. Fourth, the proposed gene signature was derived from transcriptomic associations and evaluated using statistical modeling. Although RT-qPCR validation supports the reproducibility of the panel, the biological roles of these genes in KBD remain to be established experimentally. Accordingly, the transcription factor analysis, immune association analysis, and pathway enrichment results should be regarded as exploratory rather than mechanistic. Finally, the clinical applicability of the model remains to be established. Although the model showed cross-cohort discriminatory ability, its performance, calibration, and decision thresholds may vary across populations and clinical settings. Prospective studies with standardized sampling strategies and clearly defined clinical endpoints will be necessary before the model can be considered for practical use.

## 5 Conclusion

This study identified a four-gene blood-based signature (*C4B*, *AQP1*, *HBA2*, and *ACSL6*) associated with Kashin–Beck disease using a multi-cohort transcriptomic and machine learning framework. The model demonstrated consistent discriminatory performance across independent blood cohorts and retained partial classification capacity in cartilage tissue, suggesting potential cross-tissue relevance. Integrated analyses of transcriptional regulation, immune cell composition, and pathway enrichment provided preliminary biological context for the identified gene panel. Overall, these findings suggest that this four-gene signature may serve as a candidate minimally invasive biomarker for Kashin–Beck disease. However, given the limitations of sample size and retrospective datasets, further validation in larger, prospective cohorts is required to confirm its clinical applicability and biological significance.

## Data Availability

The datasets analyzed in this study are publicly available in GEO and ArrayExpress under accession numbers GSE186593, GSE59446, GSE32127, and E-MEXP-3196. The analysis scripts used for data preprocessing, feature selection, machine-learning modeling, and model evaluation are available at https://github.com/gmh2000/KBD-four-gene-signature. Additional data generated in this study are included in the article and its **Supplementary Material**.
