# Supplementary material for: A blood-based four-gene diagnostic signature for Kashin–Beck disease revealed by multi-cohort transcriptomic analysis and machine learning
Source: Front Immunol. 2026 May 13;17:1789022. doi: 10.3389/fimmu.2026.1789022 (PMC13212447; doi:10.3389/fimmu.2026.1789022)
Supplement: Supplementary file 1 [file Image1.pdf]

(A)

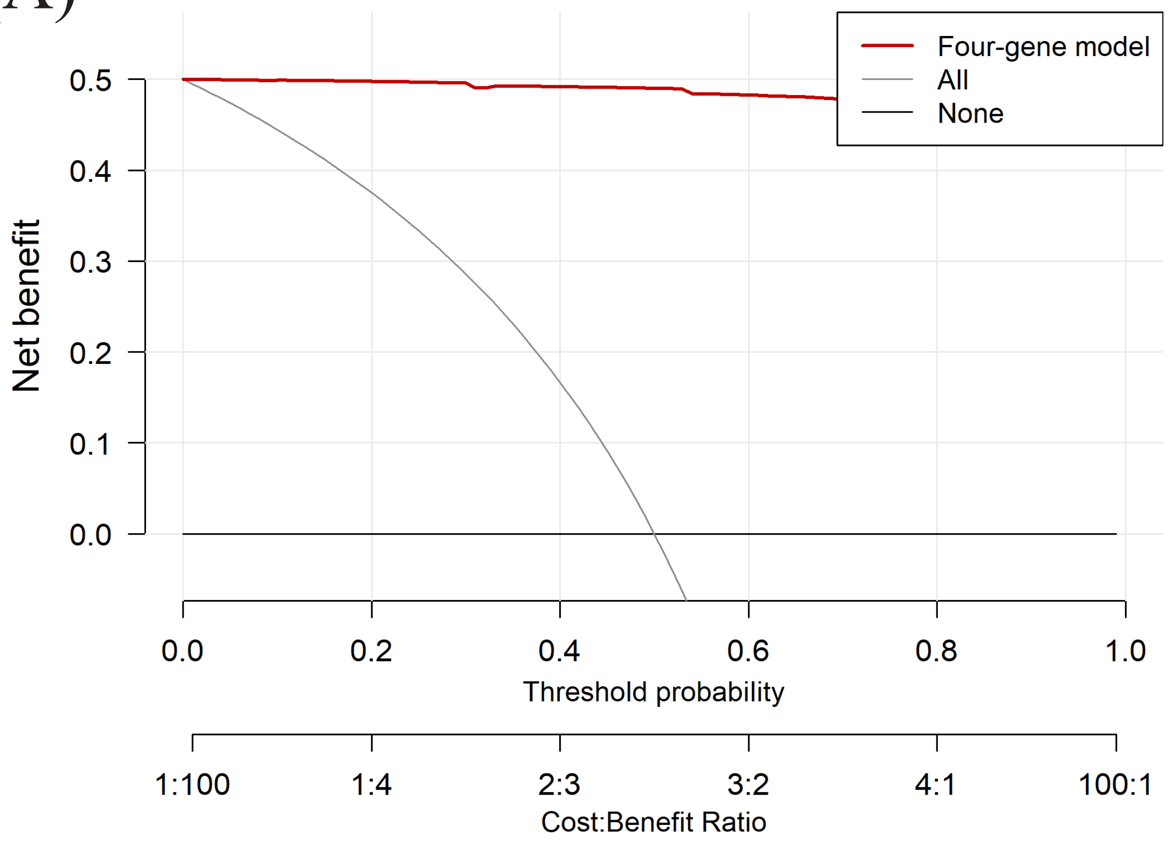

(B)

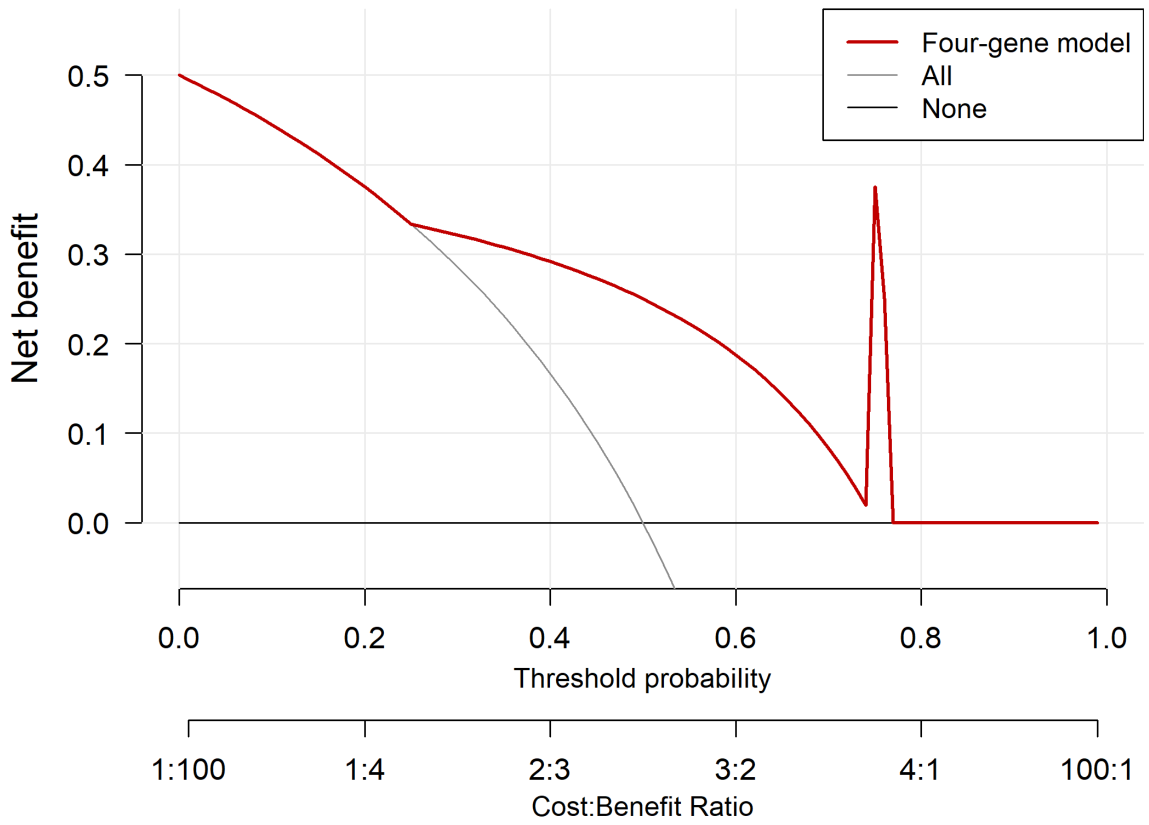

Supplementary Figure S1. Decision curve analysis of the four-gene diagnostic model, (A) Training cohort (GSE59446). The model shows a higher net benefit than the treat-all and treat-none strategies across a range of threshold probabilities, (B) Validation cohort. The model shows net benefit at lower threshold probabilities, with reduced advantage at higher thresholds.
