# Supplementary material for: A blood-based four-gene diagnostic signature for Kashin–Beck disease revealed by multi-cohort transcriptomic analysis and machine learning
Source: Front Immunol. 2026 May 13;17:1789022. doi: 10.3389/fimmu.2026.1789022 (PMC13212447; doi:10.3389/fimmu.2026.1789022)
Supplement: Supplementary file 2 [file Image2.pdf]

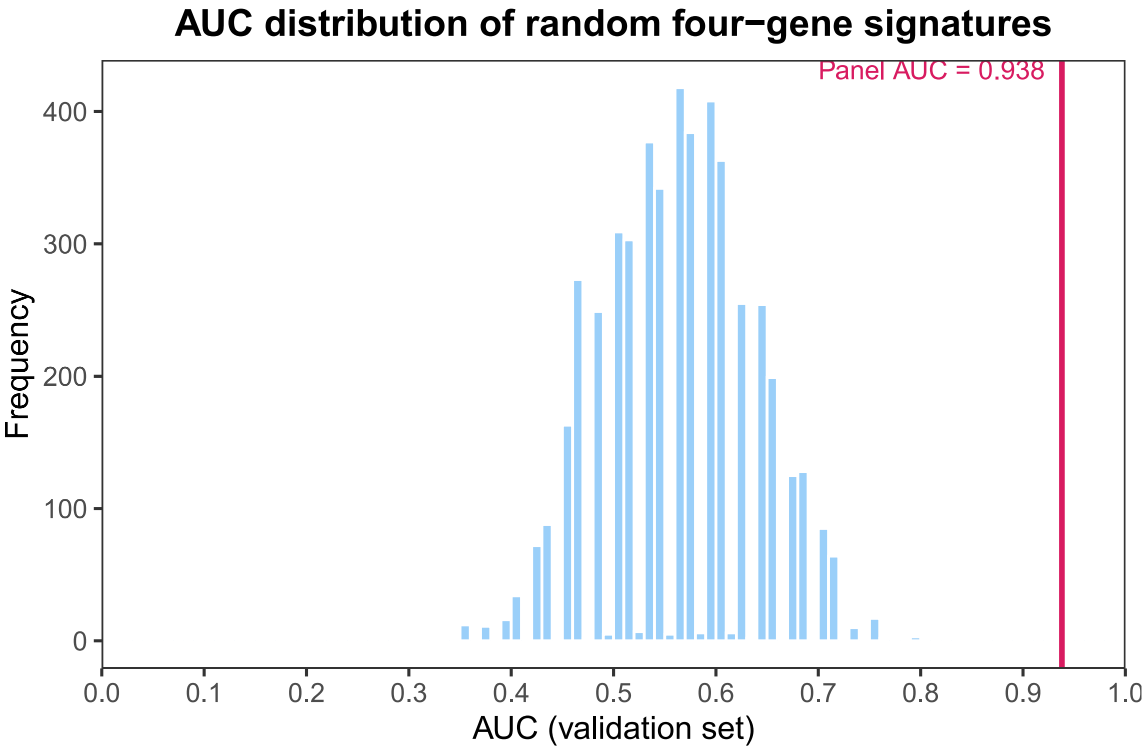

Supplementary Figure S2. AUC distribution of random four-gene signatures.
