# Supplementary material for: A blood-based four-gene diagnostic signature for Kashin–Beck disease revealed by multi-cohort transcriptomic analysis and machine learning
Source: Front Immunol. 2026 May 13;17:1789022. doi: 10.3389/fimmu.2026.1789022 (PMC13212447; doi:10.3389/fimmu.2026.1789022)
Supplement: Supplementary file 3 [file Image3.pdf]

(A)

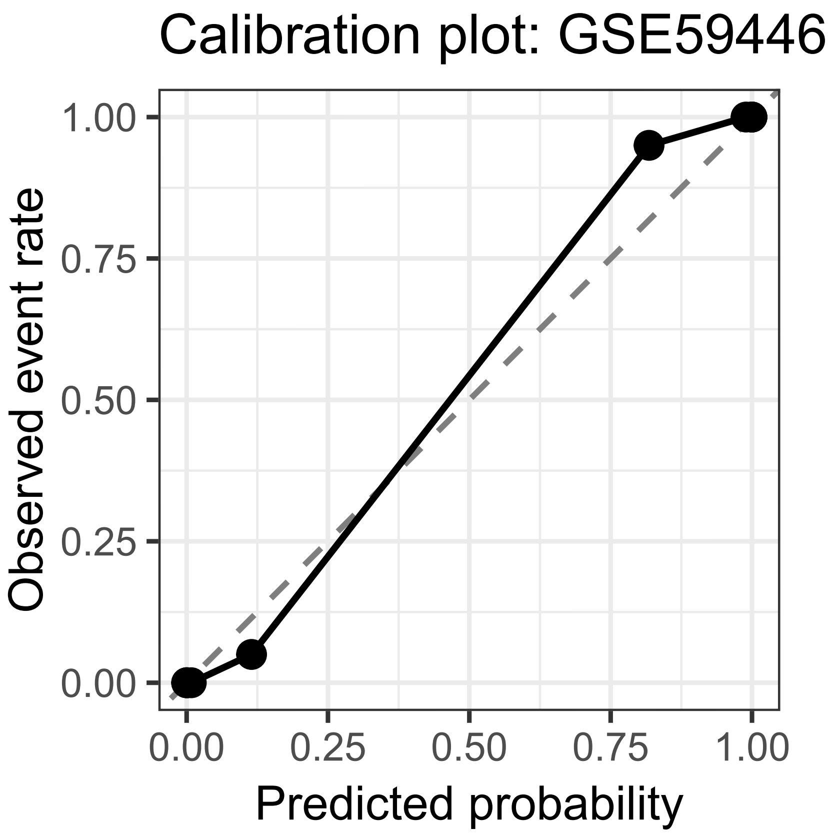

(B)

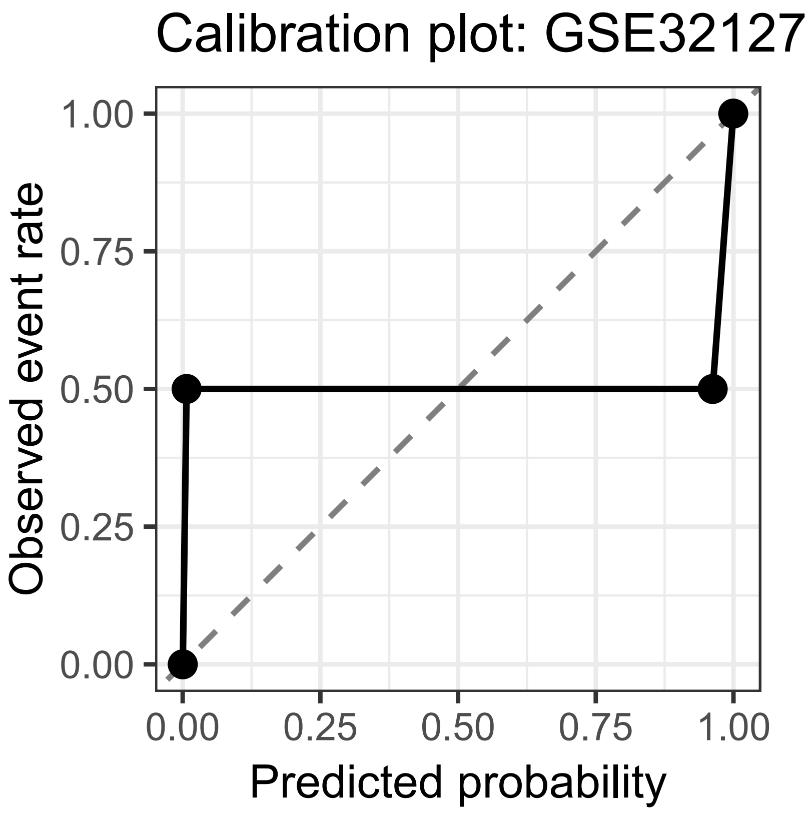

Supplementary Figure S3. Calibration analysis of the four-gene diagnostic model for Kashin–Beck disease, (A) Calibration plot of the four-gene diagnostic model in the training cohort (GSE59446), (B) Calibration plot of the four-gene diagnostic model in the external validation cohort (GSE32127).
